# Local B cells and IgE production in the oesophageal mucosa in eosinophilic oesophagitis

**DOI:** 10.1136/gut.2009.178020

**Published:** 2009-06-14

**Authors:** M Vicario, C Blanchard, K F Stringer, M H Collins, M K Mingler, A Ahrens, P E Putnam, J P Abonia, J Santos, M E Rothenberg

**Affiliations:** 1 Division of Allergy and Immunology, Department of Pediatrics, Cincinnati Children’s Hospital Medical Center, University of Cincinnati College of Medicine, Cincinnati, Ohio, USA; 2 Digestive Diseases Research Unit, Neuro-immuno-gastroenterology Lab, Department of Gastroenterology, Institut de Recerca & Hospital Universitari Vall d’Hebron, Universitat Autònoma de Barcelona, Barcelona, Spain; 3 Division of Pathology and Laboratory Medicine, Department of Pediatrics, Cincinnati Children’s Hospital Medical Center, University of Cincinnati College of Medicine, Cincinnati, Ohio, USA; 4 Division of Gastroenterology, Hepatology and Nutrition, Department of Pediatrics, Cincinnati Children’s Hospital Medical Center, University of Cincinnati College of Medicine, Cincinnati, Ohio, USA

## Abstract

**Background:**

Eosinophilic oesophagitis (EO) is an emerging yet increasingly prevalent disorder characterised by a dense and selective eosinophilic infiltration of the oesophageal wall. While EO is considered an atopic disease primarily triggered by food antigens, disparities between standard allergen testing and clinical responses to exclusion diets suggest the participation of distinct antigen-specific immunoglobulin E (IgE) in the pathophysiology of EO.

**Aim:**

To find evidence for a local IgE response.

**Methods:**

Endoscopic biopsies of the distal oesophagus of atopic and non-atopic EO and control individuals (CTL) were processed for immunohistochemistry and immunofluorescence to assess the presence of B cells, mast cells, and IgE-bearing cells. Oesophageal RNA was analysed for the expression of genes involved in B cell activation, class switch recombination to IgE and IgE production, including germline transcripts (GLTs), activation-induced cytidine deaminase (AID), IgE heavy chain (Cε) and mature IgE mRNA using polymerase chain reaction and microarray analysis.

**Results:**

Regardless of atopy, EO showed increased density of B cells (p*<*0.05) and of IgE-bounded mast cells compared to CTL. Both EO and CTL expressed μGLT, εGLT, γ4GLT, AID, Cε and IgE mRNA. However, the frequency of expression of total GLTs (p* = *0.002), εGLT (p* = *0.024), and Cε (p* = *0.0003) was significantly higher in EO than in CTL, independent of the atopic status.

**Conclusion:**

These results support the heretofore unproven occurrence of both local immunoglobulin class switching to IgE and IgE production in the oesophageal mucosa of EO patients. Sensitisation and activation of mast cells involving local IgE may therefore critically contribute to disease pathogenesis.

Eosinophilic oesophagitis (EO) is a chronic inflammatory disorder, restricted to the oesophagus, whose pathogenesis is poorly understood. Studies from North America,^1^ ^2^ Europe^3^ and Australia^4^ have indicated that EO is a growing health problem worldwide with an annual incidence of ⩾1 in 10 000.^5^ Patients with primary EO often report symptoms (chest and abdominal pain, dysphagia, heartburn, vomiting, and food impaction) also observed in gastro-oesophageal reflux disease (GORD) or chronic oesophagitis. However, in contrast to GORD patients, EO patients are predominantly males,^3^ have a familial pattern of inheritance,^6^ do not respond to treatment with proton pump inhibitors (PPIs), have normal pH monitoring,^7^ ^8^ show extensive epithelial hyperplasia and higher density of eosinophils (>15 peak eosinophils/high-power field) in the oesophageal epithelium, and over-express a unique transcriptome including the eosinophil chemokine eotaxin-3.^9^ ^10^

Several lines of evidence support the view that EO is an atopic disorder.^11^ ^12^ Most patients with EO (∼75% of cases) show signs of atopy, defined by reactivity to allergens by skin-prick testing (SPT) or identification of specific IgE in serum.^5^ ^12^ ^13^ Furthermore, EO appears to be primarily food antigen-driven since >90% of patients achieve complete remission following an elemental diet-based nutrition.^14^ However, despite the high rate of sensitisation to specific food antigens, the benefit of removing SPT-based exclusion diets is commonly unsatisfactory.^15^ ^16^ Perhaps the identification of antigen sensitisation via cutaneous testing is not reflective of the presumably IgE bound to mast cells (or basophils) in the oesophageal mucosa.

Mast cells are resident cells of the oesophagus, shown previously to be increased in number and to correlate with eosinophil counts and eotaxin-3 in the mucosa of EO patients.^10^ ^17^ In addition, the transcription of some mast cell-specific genes, such as carboxypeptidase A3, high-affinity IgE receptor (FcεR1) and tryptase-α is higher in oesophageal biopsies from patients with EO compared with healthy controls.^10^ These findings, together with the presence of mast cells in the oesophagus of patients with EO,^10^ ^17^ ^18^ ^19^ suggest a putative role for mast cell-mediated hypersensitivity in the pathogenesis of EO.

The oesophageal mucosa displays a strong immunological capacity conveyed via a diversity of resident immune cell types,^18^ ^20^ well represented by its known ability to recruit acute inflammatory cells and eosinophils.^21^ ^22^ In particular, the presence of interleukin 4 (IL4) and IL13,^22^ B cells^18^ ^19^ and of cells that are potentially CD40L^+^ (eg, T cells and mast cells) in the oesophagus supports its participation in allergen sensitisation and IgE production, highlighting a potential role for B lymphocytes and mast cells in this disease. Following antigen stimulation, mature B lymphocytes undergo class switch recombination (CSR) by changing the C region of the Ig H chain (C_H_) with a downstream region on human chromosome 14,^23^ therefore improving the antibody effector function and contributing to the maturation of the humoral response.^24^ ^25^ Induction of CSR to IgE requires mature B cells, IL4 or IL13, cognate help by CD40-L^23^ ^24^ and upregulation of activation-induced cytidine deaminase (AID).^26^ ^27^ CSR involves the transcription of germ-line genes, DNA recombination within the heavy chain locus producing ε circular transcripts, and the synthesis of immunoglobulin mature mRNA that is translated into protein.^23^ Although this phenomenon has been generally assumed to be restricted to lymphoid organs,^28^ ^29^ recent studies have demonstrated that other tissues under constant antigenic challenge such as the intestinal,^30^ ^31^ nasal^32^ and bronchial^33^ mucosa support isotype switching and IgE production.

We hypothesised that the oesophageal mucosa acts as a site for IgE generation in EO. We examined a cohort of paediatric patients and studied B cell levels in the oesophageal mucosa, and the molecular steps involved in CSR to IgE. Our results identify the human oesophagus as an immunologically active tissue with regard to B cell antibody production, suggesting a role for B lymphocytes and local IgE synthesis in EO and implicating local IgE-mediated mast cell degranulation as an important contributor to EO pathogenesis.

## Materials and methods

### Oesophageal biopsies and patient characteristics

Patients were retrospectively selected without any regard to age, atopic status, or gender from our database at the Division of Pathology and Laboratory Medicine at Cincinnati Children’s Hospital Medical Center. Biopsy specimens were collected from the distal oesophagus, fixed in formalin and processed for pathological analysis with haematoxylin & eosin (H&E) staining. Diagnosis of EO was established by a pathologist based on a maximum eosinophil count of ⩾15 eosinophils per high-power field (hpf, ×400), the presence of inflammatory infiltrate, and the hyperplasia of the basal epithelial layer. The control group (CTL) included patients with symptoms typical of GORD and EO, most of them under PPI therapy, which showed normal endoscopic and histological evaluation and the presence of ⩽5 eosinophils/hpf. We first studied the cellular infiltrate and the expression of B cell-related genes in patients from our database regardless of therapy, and thereafter selected a cohort of patients without corticosteroids or diet therapy to assess molecular markers of CSR and IgE synthesis. The detailed clinical characteristics of the selection of EO and CTL patients including eosinophil counts in the oesophageal epithelium, atopic status and therapy are shown in table 1. Patients suffering from asthma, allergic rhinitis or eczema were defined as atopic. Due to the low amount of biological material derived from endoscopic oesophageal biopsies from paediatric patients, not all samples could be processed for all experimental procedures.

**Table 1:** Clinical characteristics of patients with eosinophilic oesophagitis and of control patients

Patient no.	Age (years)/Gender	Eosinophil peak/hpf	Total IgE (ng/ml)	Asthma	Allergic rhinitis	Eczema	Therapy
Eosinophilic oesophagitis (n = 11)							
1	6/M	35	NA	+	+	−	β2-Agonist, antihistaminic
2	10/M	85	148	−	+	−	PPI, antihistaminic
3	7/M	50	20	NA	+	−	Antibiotic
4	7/M	82	NA	+	+	+	PPI, β2-agonist, antihistaminic
5	13/F	91	1590	+	+	+	PPI, β2-agonist, antihistaminic
6	4/F	28	420	−	−	+	β2-Agonist, antihistaminic
7	6/F	71	NA	−	+	−	PPI, antihistaminic
8	16/M	24	316	−	−	−	None
9	14/M	76	NA	−	−	−	PPI, LTRA
10	3/M	68	17	−	−	−	PPI
11	2/M	51	347	−	−	−	PPI
							
Controls (n = 8)							
12	2/M	0	216	+	+	−	Antibiotic
13	16/F	0	402	+	+	−	PPI
14	2/M	5	5	−	+	−	PPI, antihistaminic
15	2/M	2	16	−	+	+	PPI
16	8/M	0	NA	+	+	NA	PPI
17	7/M	2	NA	−	−	−	PPI, β2-agonist
18	8/M	3	NA	−	−	−	None
19	6/M	0	5	−	−	−	PPI, antihistaminic

Serum IgE concentration in control individuals was 96 ng/ml.^34^

F, female; hpf, high-power, field; IgE, immunoglobulin E; LTRA, leukotriene receptor antagonist; M, male; NA, not available; PPI, proton pump inhibitor.

### Immunohistochemistry

Formalin-fixed, paraffin-embedded biopsies were sectioned at 4 μm and stained with antibodies following standard procedures. B lymphocytes were identified with mouse anti-human CD20 (Dako, XXXXX, California, USA), and mast cells with mouse anti-human tryptase (Cell Marque, Rocklin, California, USA). Staining was developed with the LSAB®2 System-HRP (Dako, Carpinteria, California, USA). Sections of human tonsils and nasal polyp were used as positive control for CD20 or tryptase staining, respectively. The primary antibody was omitted as a negative control. One biopsy section from each patient was subjected to CD20 or tryptase staining. Morphometric analysis was performed for quantification of CD20^+^ cells using the Metamorph Imaging system (Universal Imaging, West Chester, Pennsylvania, USA). Total stained cells and total surface area were quantified in the three compartments of the oesophageal mucosa: the epithelium, the vascular papillae (projections of the lamina propria towards the undersurface of the epithelium)^35^ and the lamina propria, and results are expressed as number of positive cells per mm^2^ of tissue. Tryptase^+^ cells were counted in at least 10 non-overlapping fields and the results are expressed as the maximum number of positive cells per hpf.

### Immunofluorescence

Double immunofluorescence staining was performed in side-mounted paraffin sections. Mast cells were identified with rabbit anti-human CD117 (Cell Marque) and IgE-bearing cells with chicken anti-human IgE (Genway Biotech, San Diego, California, USA) primary antibodies. Secondary antibodies were Alexa Fluor 488-labelled goat anti-rabbit (Invitrogen, Carlsbad, California, USA) and biotinylated donkey anti-chicken (Jackson Labs, West Grove, Pennsylvania, USA), following Alexa Fluor 594 labelled streptavidin (Invitrogen). Slides were cover-slipped using antifade medium containing 4′,6-diamidine-2′-phenylindole dihydrochloride (DAPI) (Prolong Gold; Invitrogen) and assessed and photographed using an RT Slider digital camera (Diagnostic Instruments, Sterling Heights, Michigan, USA) mounted on an E600 fluorescence microscope (Nikon Instruments, Melville, New York, USA). Nasal polyp biopsy sections from an allergic patient were used as positive control tissue. Isotype-matched control antibodies were used as negative control. Results are expressed as the maximum number of stained cells per reticle area using a Nikon CF1 ×10 eyepiece.

### RNA isolation and DNA microarray analysis

Each biopsy from the distal oesophagus was immediately immersed in RNAlater (Qiagen, Germantown, Maryland, USA) and stored at 4°C. Total mRNA was isolated using the RNAeasy Mini Kit (Qiagen) and hybridisation to DNA microarray was performed by the Microarray Core. Microarray analysis and assessment of transcripts from B lymphocyte-related genes were performed as previously described.^36^

### Reverse transcription polymerase chain reaction and PCR analysis

The RNA samples (500 ng) were subjected to reverse transcription analysis using Inscript reverse transcriptase (Bio-Rad Laboratories, Hercules, California, USA) following the manufacturer’s instructions. Germ-line transcripts (GLTs) from εGLT, μGLT, γ1GLT and γ4GLT, and AID mRNA were amplified with validated primer sets.^32^ Mature IgE mRNA was amplified with primers JH2^37^ and CεR (5′-CAGGACGACTGTAAGATCTTCACG). To assure the integrity and to control the load of cDNA, glyceraldehyde-3-phosphate dehydrogenase was amplified with primers GAPDH-F (5′-TGGAAATCCCATCACCATCT) and GAPDH-R (5′-GTCTTCTGGGTGGCAGTGAT). PCR was run in a 23 μl reaction in the ready-to-use Supermix PCR mixture (Invitrogen) containing 3 μl of template and the corresponding primers at 0.22 μmol/l. For AID amplification, the two rounds of PCR were: denaturation at 96°C for 10 min, following 25 cycles of denaturation at 96°C for 1 min, annealing at 60°C for 1 min and elongation at 72°C for 2 min, and a final extension at 72°C for 5 min. Similar conditions were used to amplify GLT and IgE mRNA, with 30 s time on each step of the 50 or 45 PCR cycles, respectively, and annealing temperature of 60°C for εGL, 55°C for μGLT, γ1GLT and γ4GLT, and 57°C for IgE mRNA. The human Burkitt’s lymphoma B cell line Ramos (RA1; ATCC, Manassas, Virginia, USA) was used as positive control for AID and for εGLT expression, the later after stimulation with IL4 (10 ng/ml) and soluble CD40 ligand (5 mg/ml) for 48 h. The human IgE-bearing cell line U266B1 (ATCC) was used as positive control for IgE mRNA. Nuclease-free water was used as negative control.

### Real-time PCR analysis

IL4, IL13 and the heavy chain of the IgE gene (Cε) were first amplified by PCR and sequenced to confirm their identity. PCR products were used to build a standard curve for the real-time PCR reaction by means of the LightCycler instrument and LightCycler FastStart DNA Master SYBR Green I as a ready-to-use reaction mix (Roche Diagnostics, Indianapolis, Indiana, USA). Primers used were: IL4F (5′-ACATCTTTGCTGCCTCCAA), IL4R (5′-AGGCAGCGAGTGTCCTTCT); IL13F (5′-ACAGCCCTCAGGGAGCTCAT), IL13R (5′-TCAGGTTGATGCTCCATACCAT); CεF (5′-CACGCTCTCTGGTCACTATG) and CεR (see above). Amplification conditions were denaturation at 96°C for 10 min, followed by 40 cycles of denaturation at 96°C for 5 s, annealing at 60°C (IL4 and IL13), or 65°C (Cε) for 15 s and elongation at 72°C for 15 s. The expression of the different transcripts was normalised to GAPDH, and results are expressed as fold induction with respect to the CTL group.

### Statistical analysis

All histological samples were randomly coded, and sections were counted blinded, independent of the clinical protocol. The χ^2^ test was used for comparison of frequencies of GLTs and IgE transcripts. Numeric variables were analysed with the non-parametric Mann–Whitney U test. Correlation between CD20^+^ cells and tryptase^+^ counts was analysed using the Spearman rank correlation test. A value of p*<*0.05 was considered statistically significant.

## Results

### B lymphocytes and mast cells in the oesophageal mucosa

**Figure 1:**
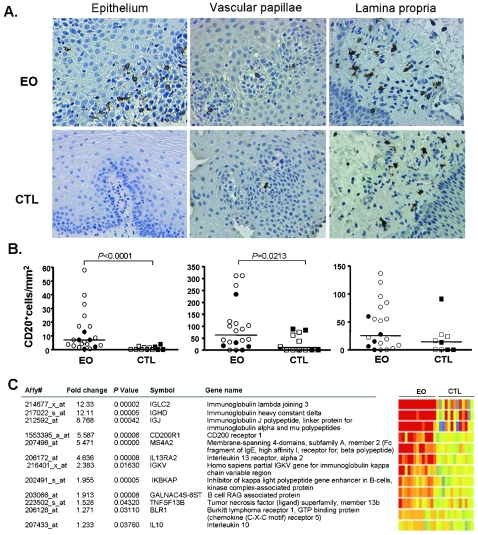
Detection of B cells in the oesophageal mucosa. (A) Representative micrographs of the CD20 staining in the epithelium, the vascular papillae and the lamina propria of the oesophageal biopsies of EO and CTL subjects. (B) Density of B cells in each compartment of the oesophageal biopsies of EO (n = 21) and CTL (n = 12) groups. Atopic (open symbols) and non-atopic patients (dark symbols) are indicated. The lines represent the median value in each group. Groups were compared using the non-parametric Mann–Whitney U test and p values are indicated. (C) Microarray analysis of the transcripts of upregulated B cell-related genes in the oesophageal biopsies of EO (n = 13) and CTL (n = 13) patients. RNA from each patient was subjected to chip analysis using Affymetrix Human Genome U133 Plus 2.0 Gene Chips. Affymetrix accession number of genes and their fold change expression in EO with respect to CTL are indicated. Statistical analysis was performed using the Welch T test; p values are indicated (n = 13 for EO and CTL groups). CTL, control; EO, eosinophilic oesophagitis.

**Figure 2:**
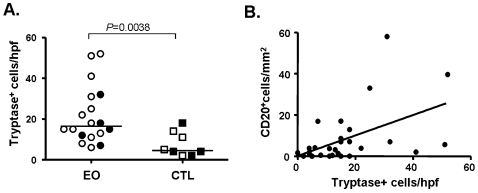
Mast cell counts in the oesophageal epithelium of EO and CTL patients. (A) Atopic (open symbols) and non-atopic patients (dark symbols) are indicated. The lines represent the median value in each group. The maximum cell count per hpf in each group was compared using the Mann–Whitney U test and the p value is indicated (EO n = 18; CTL n = 8). (B) Correlation between density of B cells and mast cells counts in the oesophageal epithelium of EO and CTL patients was calculated with the Spearman rank correlation test (p* = *0.0015; r^2^ = 0.554). Different units in each axis derive from the different methodology to assess cell counts. CTL, control; EO, eosinophilic oesophagitis.

The presence of B cells was rarely detected in the epithelium of CTL patients. However, the oesophageal epithelium of EO patients showed significantly higher density of scattered B lymphocytes, mainly in the peripapillary area, compared to CTL patients. The vascular papillae of EO samples also showed more CD20^+^ cells than the CTL samples, and no difference was observed in the lamina propria (fig 1A,B). Interestingly, atopic and non-atopic patients followed similar distribution within the EO and CTL groups. Furthermore, microarray analysis revealed that EO patients have higher expression of B cell-related genes than CTL subjects (fig 1C) including immunoglobulin lambda joining 3, immunoglobulin heavy constant delta, immunoglobulin J polypeptide and B cell RAG-associated protein (with a 12.3-, 12.1-, 8.8- and 1.9-fold increase, respectively (p*<*0.001)). Mast cell infiltration was also increased in EO compared to CTL samples (fig 2), as previously shown^10^ ^18^ ^19^ with similar distribution of atopic and non-atopic subjects within each group. Notably, the density of CD20^+^ cells positively correlated with mast cells (p* = *0.0015; r^2^ = 0.554; fig 2B), but not with eosinophils in the epithelium (data not shown). Positive control staining procedure for human tonsils or nasal polyp revealed the presence of B cells and mast cells, respectively, and negative control procedure did not detect any positive cells (data not shown).

### Expression of IL4 and IL13

**Figure 3:**
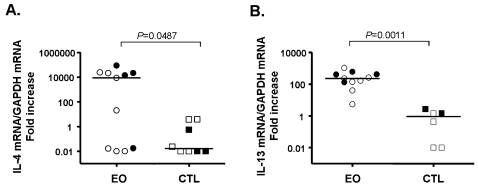
Analysis of the expression of IL4 (A) and IL13 (B) in oesophageal biopsies from EO and CTL patients. Atopic (open symbols) and non-atopic patients (dark symbols) are indicated. Each mRNA value was normalised to GAPDH mRNA expression from the same cDNA and is expressed as fold change with respect to the CTL group. The lines represent the median value in each group. EO (n = 11) and CTL (n = 6−8) groups were compared using the Mann–Whitney U test. Values of p are indicated. CTL, control; EO, eosinophilic oesophagitis; GAPDH, glyceraldehyde-3-phosphate dehydrogenase; IL, interleukin.

Real-time PCR demonstrated that IL4 mRNA expression was increased in EO compared with CTL, particularly in a subset of EO patients (fig 3A), with no difference related to atopy, and that IL13 mRNA was over-produced in EO, as previously shown.^22^ Interestingly, atopic and non-atopic EO patients had comparable levels of IL13 mRNA (fig 3B).

### Expression of GLT in the oesophageal mucosa

**Figure 4:**
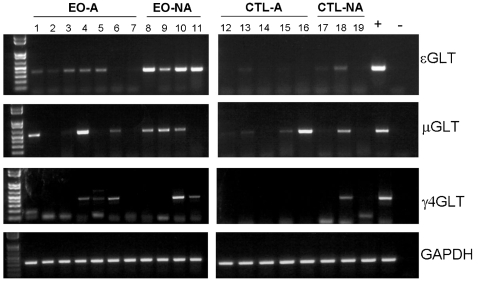
Analysis of the expression of GLTs in oesophageal biopsies of EO and CTL patients. Patients are divided into atopic (EO-A, n = 7; CTL-A, n = 5) and non-atopic (EO-NA, n = 4; CTL-NA, n = 3) groups. The εGLT, 379 bp; μGLT, 529 bp; and γ4GLT, 505 bp bands were PCR amplified from oesophageal biopsies of EO and CTL patients. GAPDH was amplified to control cDNA loading. Lanes + and − correspond to PCR positive and negative controls, respectively. A 1 kb plus DNA ladder was loaded in all cases. Gels are representative of one of three independent PCR amplifications yielding similar results. CTL, control; EO, eosinophilic oesophagitis; GAPDH, glyceraldehyde-3-phosphate dehydrogenase; GLTs, germ-line transcripts; PCR, polymerase chain reaction.

The transcription of GLTs is essential for CSR and is the first step in the commitment of B cells to the synthesis of IgG, IgA and IgE. We detected εGLT, μGLT and γ4GLT (fig 4) but not αGLT, γ1GLt or γ3GLT (data not shown) in oesophageal biopsies. The frequency of expression of total GLTs was higher in EO patients (p* = *0.012), being individually nine of 11 EO, and three of eight CTL for εGLT (p* = *0.024); six of 11 EO, and four of eight CTL for μGLT (p* = *0.456); and five of 11 EO, and one of eight CTL for γ4GLT (p* = *0.064). Interestingly, GLTs were similarly detected in both atopic and non-atopic EO patients. All PCR products were gel-extracted and sequenced to confirm their identity with the corresponding IgH chain sequences present in GeneBank (data not shown).

### Expression of AID in the oesophageal mucosa

**Figure 5:**
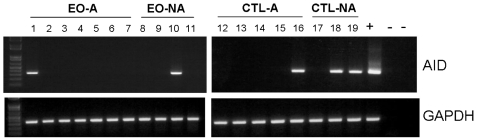
Analysis of the expression of AID in oesophageal biopsies of EO and CTL patients. Patients are divided into atopic (EO-A, n = 7; CTL-A, n = 5) and non-atopic (EO-NA, n = 4; CTL-NA, n = 3) groups. The 335 bp AID mRNA band was amplified in a semi-nested PCR. GAPDH was amplified to control cDNA loading. Lanes + and − correspond to PCR positive and negative controls, respectively. The two negative – lanes indicate first-round and second-round PCR-negative controls. A 1 kb plus DNA ladder was loaded. Gels are representative of one of three independent PCR amplifications yielding similar results. AID, activation-induced cytidine deaminase; CTL, control; EO, eosinophilic oesophagitis; GAPDH, glyceraldehyde-3-phosphate dehydrogenase; PCR, polymerase chain reaction.

The enzyme AID catalyses the initial step of CSR in germinal-centre B cells, and has recently been detected outside lymphoid structures.^32^ ^33^ Here, we detected AID expression in biopsies from both groups, indicating the potential of the oesophageal mucosa to undergo CSR (fig 5). PCR products from all samples were sequenced and identified as human AID mRNA (data not shown).

### Expression of IgE heavy chain mRNA in the oesophageal mucosa

**Figure 6:**
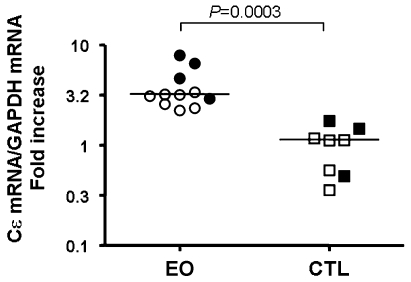
Analysis of the expression of Cε by real-time PCR in the oesophageal biopsies of EO and CTL patients. Atopic (open symbols) and non-atopic patients (dark symbols) are indicated. Each mRNA value is normalised to GAPDH mRNA expression from the same sample and is expressed as fold increase compared to the CTL group. The lines represent the median value in each group. EO (n = 11) and CTL (n = 8) groups were compared using the Mann–Whitney U test. The p value is indicated. CTL, control; EO, eosinophilic oesophagitis; GAPDH, glyceraldehyde-3-phosphate dehydrogenase; PCR, polymerase chain reaction.

All EO subjects showed higher expression of Cε than CTL patients, as determined by quantitative PCR (fig 6). The amplified PCR product is representative of either the sterile transcripts or the mature IgE mRNA, and is increased in biopsies from both atopic and non-atopic EO patients. These data confirm, at least at the mRNA level, that local IgE expression is a feature of the oesophageal mucosa of EO, regardless of the atopic status.

### Expression of mature IgE mRNA in the oesophageal mucosa

**Figure 7:**
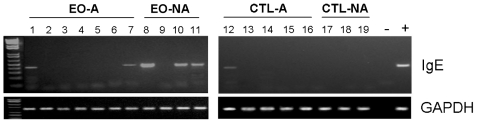
Analysis of the expression of mature IgE mRNA in oesophageal biopsies of EO and CTL patients. Patients are divided into atopic (EO-A, n = 7; CTL-A, n = 5) and non-atopic (EO-NA, n = 4; CTL-NA, n = 3) groups. The 412 bp IgE band was PCR amplified from oesophageal biopsies of EO and CTL patients, and GAPDH was amplified to control cDNA loading. Lanes − and + correspond to PCR negative and positive controls, respectively. A 1 kb plus DNA ladder was loaded. Gels are representative of one of three independent PCR amplifications yielding similar results. CTL, control; EO, eosinophilic oesophagitis; GAPDH, glyceraldehyde-3-phosphate dehydrogenase; PCR, polymerase chain reaction.

The final step of CSR is antibody production. Mature IgE mRNA was detected in five of 11 EO, regardless of atopy, and in one of eight CTL subjects (fig 7). Differences in the frequency of expression did not reach statistical significance between EO and CTL (p* = *0.0635). Importantly, and consistent with the data presented herein, detection of mature IgE mRNA suggests in situ production of IgE in the oesophageal mucosa, independent of the atopic status of the patient.

### Detection of IgE-bearing cells in the oesophageal epithelium

**Figure 8:**
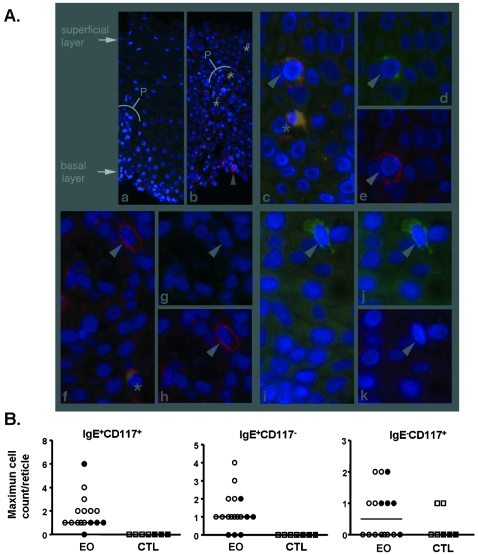
Detection of IgE^+^ and CD117^+^ cells in the oesophageal epithelium by double immunofluorescence. (A) Representative micrographs of the oesophageal epithelium where IgE-bearing cells (red) and mast cells (green) are identified (arrow head). An example of CTL and EO biopsies (a and b micrographs, respectively) is shown, where epithelial hyperplasia, elongation of the vascular papillae (P) and eosinophils (asterisk) are detected (low-power field, ×200). The three identified populations are shown in groups of three micrographs each, at high-power field (×600): IgE^+^CD117^+^ (c, d, e); IgE^+^CD117^−^ (f, g, h); IgE^−^CD117^+^ (i, j, k). (B) Quantification of the cell populations based on IgE and CD117 positivity in the oesophageal epithelium of EO (n = 16) and CTL (n = 7) patients. The maximum number of positive cells per reticle is represented in each group. Atopic (open symbols) and non-atopic patients (dark symbols) are indicated. CTL, control; EO, eosinophilic oesophagitis.

We detected three different cell populations in the oesophageal epithelium based on IgE and CD117 positivity (fig 8). IgE^+^ cells were detected only in EO and mast cells were present in both groups; however, only the EO group showed IgE-bearing mast cells. Of the total intraepithelial mast cells, the percentage of IgE^+^ cells was increased in atopic compared to non-atopic EO patients (85.6%; 95% CI, 70 to 100; and 43.8%, 95% CI 5.2 to 82, respectively, p*<*0.05). Notably, the epithelium contained a population of IgE^+^ CD117^−^ cells which was absent in CTL subjects.

## Discussion

Eosinophilic oesophagitis is a chronic inflammatory disease in which the presence of mediators such as IL5, IL13 and eotaxin, the cellular infiltrate (eosinophils, mast cells and lymphocytes), and the association with allergic disorders, all point to a Th2-associated disease. A prominent feature of the Th2 immune response is antibody production; however, the participation of B cells in the pathogenesis of EO has not been extensively studied, probably because of the low amount of infiltrating B cells as compared with other immunocytes in the oesophageal epithelium. Our study also shows that B lymphocytes are moderately increased, consistent with studies in adult EO patients,^18^ ^19^ suggesting a similar pathogenic process in both adults and children. Notably, since B cell infiltration may mainly occur through the vascular supply of the epithelium, the increase in B lymphocytes in the vascular papillae, consistent with that of the epithelium, suggests that B cell recruitment is an active process in the oesophageal mucosa in EO disease. Furthermore, differential expression of immunoglobulin genes and other B cell genes in EO compared to CTL subjects, together with IgE production in the oesophageal mucosa, highlight B cells as important contributors to EO pathogenesis.

CSR has been restricted to lymphoid tissues; however, recent studies have proved this phenomenon at certain mucosal sites.^32^ ^33^ The present study has discovered the expression of GLT, AID, Cε and IgE mRNA in the human oesophagus, providing the first evidence for local class switching to IgE and IgE production, and actually indicating that the oesophageal mucosa is primed for switching to IgE in EO. GLT expression demonstrates the potential of B cells to undergo CSR, but it does not provide definitive evidence that switching has actually occurred.^38^ In order to confirm the ongoing process, the presence of either switch circles or circular transcripts needs to be demonstrated.^27^ ^39^ However, since none of those molecular markers were detected in EO biopsies (data not shown), we cannot exclude the possibility that B cells switched elsewhere and migrated to the oesophagus. Nevertheless, the expression of switch circles and circular transcripts is transient, and they are not detected unless switching has taken place recently.^38^ In order to detect ongoing switching, it may be necessary to collect biopsies precisely at the time of exposure to the allergen, as has been done in rhinitis patients during the allergy season.^32^ In our study, specimen collection was performed during the course of the disease, and did not take into account whether patients were avoiding exposure to specific foods prior to the endoscopy; indeed, as part of the preparation, patients are fasted overnight. This may explain why switch circles or circular transcripts were undetectable in our samples. According to the hypothesis that switching already occurred when biopsies were collected, CD40L was not upregulated in EO (data not shown), as it is rapidly and transiently expressed on recently stimulated T helper cells and antigen-presenting cells upon activation.^40^ ^41^

Atopy is more prevalent in subjects who have EO than in the general population.^6^ ^12^ Clinical studies have shown a high prevalence of atopic diseases in EO patients.^6^ ^13^ In our study, rhinitis had the highest prevalence among atopic manifestations (55%), highlighting a possible link between the immune response in the respiratory tract and the oesophageal mucosa. Notably, intranasal, but not oral or intragastric allergen challenge induces experimental EO,^42^ suggesting a role for inhaled allergens. Nonetheless, not all EO patients have clinical manifestations of atopy or show positivity to SPT and this fact led us to analyse the Th2 response in atopic and non-atopic EO patients and to compare it with the same two groups in CTL patients. Interestingly, the recently identified EO transcript signature has been proven to be remarkably conserved between patients despite their age, sex, familial inheritance pattern and allergic status, suggesting that allergic and non-allergic variants of EO have a common underlying pathogenesis.^6^ ^10^ Those results imply that the present study’s findings of accumulation of B cells expressing molecular markers of CSR and detection of IgE production would be comparable across both clinically defined atopic and non-atopic EO patients. Indeed, and importantly, perhaps “non-atopic” EO patients are actually atopic, but to a lesser extent.

IL4 and IL13 have a key role in the induction of IgE switching by stimulating transcription from the germline promoter site of IgE, via STAT6 sites,^23^ and are also B cell proliferating factors.^43^ Here we confirmed over-expression of IL13 mRNA in EO^22^ and demonstrated that transcription of IL13 is not influenced by the atopic status of the patient. We have previously shown that the expression of IL4 in EO patients is similar to control patients although there was great heterogeneity in levels,^22^ perhaps because, in that previous study, patients with EO were selected regardless of therapy. However, when selecting non-corticosteroid and non-dietary treated EO patients, IL4 mRNA proved higher in EO than in CTL patients. Importantly, the participation of IL4 in promoting Th2 responses and stimulating class switching to IgG4 and IgE^23^ ^44^ is suggested here, as demonstrated by the expression of εGLT and γ4GLT in EO subjects.

The presence of IgE^+^ cells in the oesophageal epithelium has been shown previously in EO, and it has been assumed that those IgE-bearing cells were all mast cells.^17^ ^18^ ^19^ Herein, we demonstrate that IgE-sensitised mast cells are a feature of the EO epithelium, and also that the percentage of mast cells linked to IgE is increased in atopic compared to non-atopic EO patients. Mechanisms other than antigen cross-linking of IgE on the surface also activate mast cells to release mediators. Of importance to EO, eosinophils and mast cells both secrete mediators relevant for mutual activation and survival.^45^ In our study, two populations of intraepithelial mast cells were detected based on IgE surface positivity, suggesting different coexisting mechanisms of activation of mast cells in EO. Notably, the correlation between CD20^+^ cells and tryptase^+^ cells, the expression of IgE mRNA and the density of IgE^+^CD117^+^ in EO, suggest that IgE-mediated mast cell activation importantly contributes to disease pathogenesis.

Of interest, we report a population of IgE-bearing cells lacking CD117 expression. Eosinophils could account for this population, since they express FcεRI and FcεRII.^46^ However, they are highly autofluorescent, and observation under a fluorescence microscope confirmed that none of them were bound to IgE. Memory B cells and plasmablasts can express IgE and are likely to represent that IgE^+^CD117^−^ population. In patients with allergic rhinitis, approximately 4% of the B cells and 15% of plasma cells express IgE in the nasal mucosa and, in that situation, it has been suggested that the maturation of IgE-expressing B cells (activated or memory) to IgE-producing plasma cells takes place locally.^47^ The presence of B cells and of IgE^+^ cells in the oesophageal epithelium has been proven previously.^17^ ^18^ ^19^ In our study, the appearance of B cells primed to isotype switch and the detection of IgE mRNA, led us to hypothesise that mature B cells are locally challenged by allergen, followed by the formation of IgE-memory B cells and IgE plasmablasts. In keeping with this, we also showed increased expression of immunoglobulin and stimulating B cell genes in EO. However, we do not exclude the possibility that already stimulated B lymphocytes at other mucosal sites infiltrate the epithelium where, upon allergen stimulation, they locally mature to IgE plasmablasts. Notably, the high rate of transcription of the IGJ gene in EO biopsies, points at plasmablasts as candidates for those IgE^+^CD117^−^ cells, since the Ig *J* chain gene is expressed only after the terminal differentiation of B cells towards plasma cells.^48^

In summary, we have demonstrated increased B cells and expression of molecular Ig machinery (eg, Ig genes and recombination enzymes) in the oesophageal mucosa of paediatric patients with EO regardless of the atopic status. Furthermore, we have determined that IgE^+^ cells (especially mast cells) are a specific feature of EO compared with control individuals. As such, we propose that the oesophageal mucosa is a site for initiation and development of humoral responses. These results offer an explanation for the dissociation between skin-prick test results and food elimination diets in EO.
